# Using high-throughput phenotype platform MVS-Pheno to reconstruct the 3D morphological structure of wheat

**DOI:** 10.1093/aobpla/plae019

**Published:** 2024-03-29

**Authors:** Wenrui Li, Sheng Wu, Weiliang Wen, Xianju Lu, Haishen Liu, Minggang Zhang, Pengliang Xiao, Xinyu Guo, Chunjiang Zhao

**Affiliations:** College of Information Engineering, Northwest A&F University, Xinong Road, Yangling, Shaanxi, Xianyang 712100, China; Information Technology Research Center, Beijing Academy of Agriculture and Forestry Sciences, Shuguang Huayuan Middle Road, Haidian District, Beijing 100097, China; Beijing Key Lab of Digital Plant, National Engineering Research Center for Information Technology in Agriculture, Shuguang Huayuan Middle Road, Haidian District, Beijing 100097, China; Information Technology Research Center, Beijing Academy of Agriculture and Forestry Sciences, Shuguang Huayuan Middle Road, Haidian District, Beijing 100097, China; Beijing Key Lab of Digital Plant, National Engineering Research Center for Information Technology in Agriculture, Shuguang Huayuan Middle Road, Haidian District, Beijing 100097, China; Information Technology Research Center, Beijing Academy of Agriculture and Forestry Sciences, Shuguang Huayuan Middle Road, Haidian District, Beijing 100097, China; Beijing Key Lab of Digital Plant, National Engineering Research Center for Information Technology in Agriculture, Shuguang Huayuan Middle Road, Haidian District, Beijing 100097, China; Information Technology Research Center, Beijing Academy of Agriculture and Forestry Sciences, Shuguang Huayuan Middle Road, Haidian District, Beijing 100097, China; Beijing Key Lab of Digital Plant, National Engineering Research Center for Information Technology in Agriculture, Shuguang Huayuan Middle Road, Haidian District, Beijing 100097, China; Information Technology Research Center, Beijing Academy of Agriculture and Forestry Sciences, Shuguang Huayuan Middle Road, Haidian District, Beijing 100097, China; Beijing Key Lab of Digital Plant, National Engineering Research Center for Information Technology in Agriculture, Shuguang Huayuan Middle Road, Haidian District, Beijing 100097, China; Information Technology Research Center, Beijing Academy of Agriculture and Forestry Sciences, Shuguang Huayuan Middle Road, Haidian District, Beijing 100097, China; Beijing Key Lab of Digital Plant, National Engineering Research Center for Information Technology in Agriculture, Shuguang Huayuan Middle Road, Haidian District, Beijing 100097, China; Information Technology Research Center, Beijing Academy of Agriculture and Forestry Sciences, Shuguang Huayuan Middle Road, Haidian District, Beijing 100097, China; Beijing Key Lab of Digital Plant, National Engineering Research Center for Information Technology in Agriculture, Shuguang Huayuan Middle Road, Haidian District, Beijing 100097, China; Information Technology Research Center, Beijing Academy of Agriculture and Forestry Sciences, Shuguang Huayuan Middle Road, Haidian District, Beijing 100097, China; Beijing Key Lab of Digital Plant, National Engineering Research Center for Information Technology in Agriculture, Shuguang Huayuan Middle Road, Haidian District, Beijing 100097, China; College of Information Engineering, Northwest A&F University, Xinong Road, Yangling, Shaanxi, Xianyang 712100, China; Information Technology Research Center, Beijing Academy of Agriculture and Forestry Sciences, Shuguang Huayuan Middle Road, Haidian District, Beijing 100097, China

**Keywords:** 3D reconstruction, plant morphology, point cloud segmentation, Wheat

## Abstract

It is of great significance to study the plant morphological structure for improving crop yield and achieving efficient use of resources. Three dimensional (3D) information can more accurately describe the morphological and structural characteristics of crop plants. Automatic acquisition of 3D information is one of the key steps in plant morphological structure research. Taking wheat as the research object, we propose a point cloud data-driven 3D reconstruction method that achieves 3D structure reconstruction and plant morphology parameterization at the phytomer scale. Specifically, we use the MVS-Pheno platform to reconstruct the point cloud of wheat plants and segment organs through the deep learning algorithm. On this basis, we automatically reconstructed the 3D structure of leaves and tillers and extracted the morphological parameters of wheat. The results show that the semantic segmentation accuracy of organs is 95.2%, and the instance segmentation accuracy AP_50_ is 0.665. The *R*^2^ values for extracted leaf length, leaf width, leaf attachment height, stem leaf angle, tiller length, and spike length were 0.97, 0.80, 1.00, 0.95, 0.99, and 0.95, respectively. This method can significantly improve the accuracy and efficiency of 3D morphological analysis of wheat plants, providing strong technical support for research in fields such as agricultural production optimization and genetic breeding.

## Teaser

We propose a point cloud data-driven 3D wheat reconstruction method, which achieves plant-scale 3D structure reconstruction and wheat morphological parameterization. First, we used the MVS-Pheno platform to reconstruct the 3D point cloud of wheat and then performed instance segmentation of the wheat organ point cloud based on the deep learning method. On this basis, we automatically reconstructed the 3D structure of the leaves and tillers and extracted the wheat morphological parameters. This method can significantly improve the accuracy and efficiency of 3D morphological analysis of wheat plants.

## Introduction

Wheat, as one of the most important grain crops in the world, has an increasingly acute contradiction between supply and demand. The increase in wheat yield is directly related to international food security. The wheat plant morphological structure is closely related to yield, and a reasonable plant morphological structure can significantly improve crop yield and economic efficiency. Shaping an ideal plant type is one of the vital breakthrough directions in breeding new wheat varieties (Liu *et al.* 2021; Liu *et al.* 2022). 3D reconstruction of wheat plants is a crucial step for digitizing and quantifying the wheat morphological structure and optimizing the design of the ideal plant type (Chang *et al.* 2023). The research on the functional–structural plant model of wheat relies on detailed 3D morphological data of its unit structure (Vos *et al.* 2010; Wang *et al.* 2023). Wheat is a multi-tillering plant with complex structural characteristics such as narrow and long leaves. Traditional manual measurement methods can obtain data on wheat plant structural units, but there are many disadvantages, such as heavy workload, low efficiency, incomplete representation of the plant’s 3D structure, and interference from subjective human factors (level of operational experience, handling methods and recording errors) (Yang *et al.* 2021). Therefore, how to accurately and automatically obtain 3D data and construct a 3D morphological structure model of wheat plants has become a critical problem that needs to be solved urgently (Zhao *et al.* 2019).

In terms of plant 3D modelling, there are four main categories: rule-based methods (Hidayat *et al.* 2020; Cieslak *et al.* 2022; Yang *et al.* 2022), 3D digitizer-based methods (Wang *et al.* 2018; Chenxi *et al.* 2022), image-based methods (Lou *et al.* 2014; Wu *et al.* 2018; Das Choudhury *et al.* 2020; Lu *et al.* 2021; Wu *et al.* 2022) and laser scanning-based methods (Ma *et al.* 2019; Sun and Wang 2019; Jin *et al.* 2021; Sun *et al.* 2021). Among them, the rule-based method can realize the 3D growth modelling of plants by specifying rules and parameterization. The classical methods mainly include the L-system (Lindenmayer 1968), reference axis technology (de Reffye *et al.* 1988) and fractal method (Oppenheimer 1986). This method was mainly used to carry out the research on functional–structural plant models in the early stage. However, the limitation of this method is that it is difficult to simulate the plant morphology with complex branching structure accurately, there is a large deviation from the actual growth situation, and it is difficult to reflect the variety of characteristics of plants. The method based on 3D digitization mainly obtains the skeleton key nodes of plant leaves, stems, and other organs through the 3D digitizer and carries out 3D reconstruction on this basis. This method has high accuracy, but it also has some shortcomings, such as the need for manual operation, the low efficiency of data acquisition, and the large influence of human factors on data quality.

In recent years, with the development of machine vision and 3D sensor technology, depth camera, lidar and multi-view stereo reconstruction technology have played an important role in acquiring 3D information and 3D reconstruction of plants and have been widely used in various agricultural research. Depth cameras and lidar can directly obtain accurate 3D point cloud data of plants. However, this method has the disadvantages of high equipment cost, low acquisition efficiency, and difficulty in obtaining point cloud data from multiple perspectives at the same time (Saeed *et al.* 2023a). In contrast, to meet the demand for refined data acquisition at the individual plant scale and organ scale, the 3D reconstruction based on multi-view images has been proven more effective. This method uses structure from motion (SFM) (Bolles *et al.* 1987) to estimate the camera’s attitude information and reconstruct the sparse point cloud, then uses multiple view stereo (MVS) (Goesele *et al.* 2007) to estimate the dense point cloud. This method generates high-efficiency data acquisition with high point cloud density, and the plant high-throughput phenotyping platform developed based on this technology also has the advantages of low cost and easy deployment (Wu *et al.* 2022).

The 3D point cloud data of plants obtained through multi-view stereo reconstruction technology has shortcomings such as point cloud disorder, large data volume, and uneven density, making data processing difficult. The core problem of extracting 3D morphological structure information from plant point cloud data is how to segment the organs of single plant point cloud data and extract phenotypic parameters related to morphological structure at the individual and organ scales. At present, point cloud data segmentation can be divided into traditional methods and deep learning methods. The traditional point cloud segmentation methods mainly rely on the geometric constraints of the point cloud and the features extracted by statistical rules, including segmentation based on manual point cloud features (Rusu *et al.* 2009), region growth (Vo *et al.* 2015) and clustering (Shi *et al.* 2011). Deep learning methods include multi-view-based methods, voxel-based methods and point cloud-based methods (Zhang *et al.* 2019).

Li *et al.* (2020) proposed a framework that includes multi-view stereo point cloud reconstruction, preprocessing, stem removal in the canopy, leaf segmentation and leaf phenotype feature extraction, using ornamental plants *Maranta arundinacea* and *Dieffen-bachia picta* as examples. This framework can accurately extract phenotypic traits such as plant leaf area, length, width and inclination angle. Li *et al.* (2022) proposed a technology that integrates high-throughput data collection and plant point cloud segmentation based on deep learning, using corn as an example, to segment corn stems and leaves and extract phenotypic indicators such as plant height, stem length, leaf length, leaf width, leaf inclination angle and leaf growth height. Saeed *et al.* (2023b) used the PVCNN model to segment cotton plant organs and obtain important structural features. Zou *et al.* (2023) proposed a mature wheat density estimation method based on the octree segmentation algorithm, voxel grid merging algorithm and point cloud clustering and established a relationship model between the number of wheat spike point clouds and the number of wheat plants. However, most current research is limited to semantic segmentation of plant point clouds and extraction of more basic phenotype parameters without further in-depth research on the 3D structure of plants.

To solve the problem of automatic and high-precision 3D reconstruction based on data-driven, we propose a method of 3D reconstruction and plant morphological parameters extraction of wheat plants based on point cloud data-driven. First, we acquired multi-view images of a single wheat plant with multi-view image acquisition equipment MVS-Pheno and reconstructed a 3D point cloud of wheat plants using the SFM-MVS algorithm. After preprocessing the point cloud data, we build a point cloud instance segmentation deep learning model based on the SoftGroup network model to perform the segmentation of tillers, leaves and ear organs of wheat plants. Then, we modelled the wheat plant tillers and leaves based on the segmentation results to obtain a 3D skeleton model of the wheat plant. Furthermore, based on the reconstructed 3D model of the wheat plant, we extracted plant morphological parameters of the wheat leaves, tillers and ears. This method provides an efficient, fast and accurate scheme for 3D reconstruction and plant morphological parameters extraction of wheat.

## Materials and Methods

**Figure 1.**
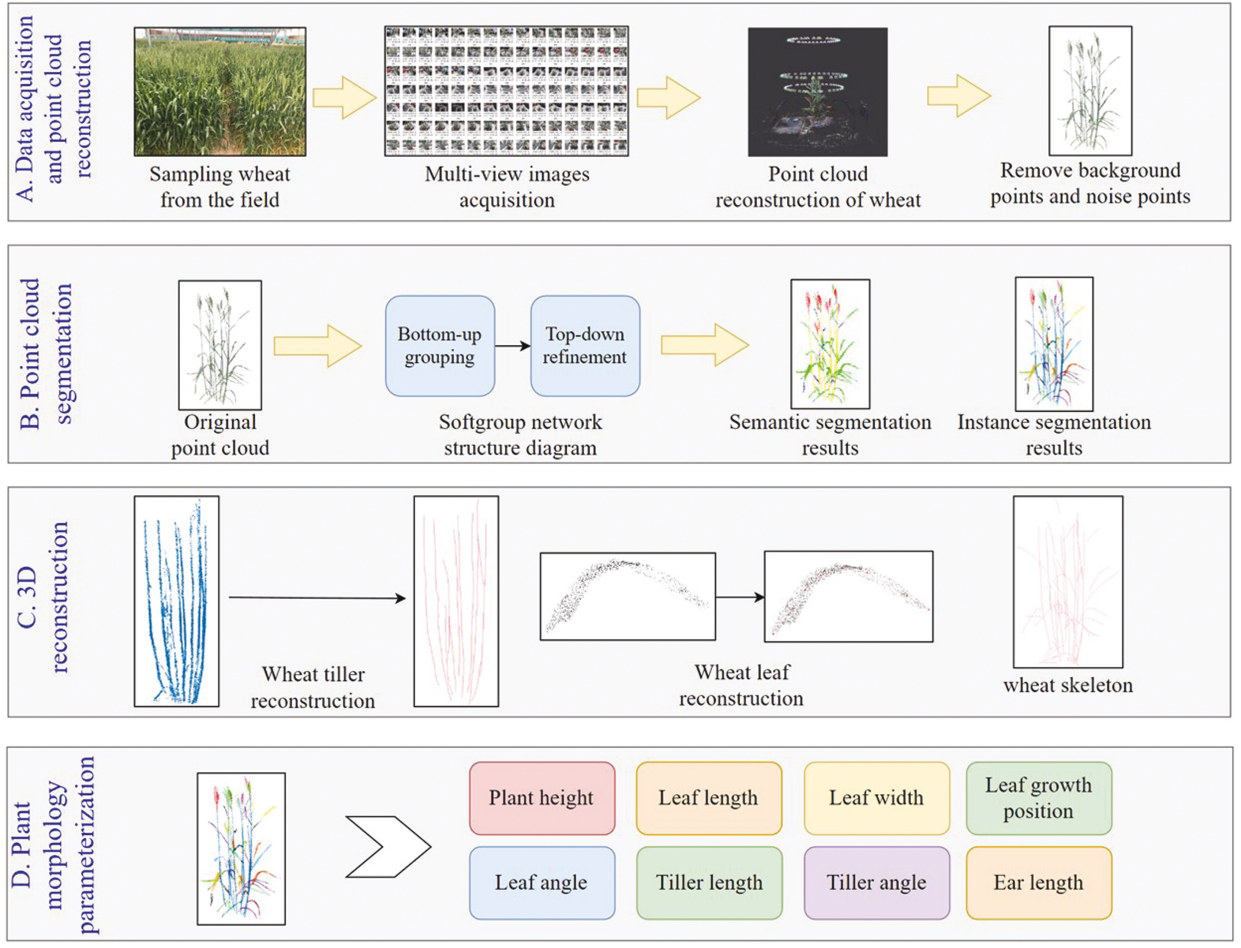
Overall flow chart of wheat 3D reconstruction and plant morphological parameters extraction. (A) High-throughput data acquisition of wheat, point cloud reconstruction, and pre-processing. (B) Wheat organ segmentation using SoftGroup network. (C) 3D reconstruction of wheat tillers and leaves. (D) Extracting plant morphological parameters of wheat based on segmentation and reconstruction results.

The method in this article mainly includes the following four parts: (i) Wheat point cloud data acquisition and data preprocessing. (Materials and Methods ii) Wheat point cloud organ segmentation based on deep learning. (iii) 3D reconstruction of wheat plants. (iv) Extraction of wheat plant morphological parameters. The overall flow chart is shown in Figure 1.

### Experimental design

The experiment was conducted in the experimental field of the Beijing Academy of Agricultural and Forestry Sciences (n 39° 560, e 116° 160) from 2020 to 2021. The experiment covered 20 varieties of winter wheat, each variety was sown in one plot (2.25 m long and 1.5 m wide) on 4 October 2020, with row spacing of 0.2 meters and plant spacing of 0.05 m. Before sowing in the field, we used the broadcast method to apply compound fertilizer (N-P2O5-K2O-12-18-15) and the fertilizer amount was 50 kg/mu, and urea was topdressing with water at the jointing stage with 20 kg/mu of fertilizer. We apply winter irrigation once during the overwintering period and once each during the jointing stage, grouting stage and maturity stage, and the amount of irrigation each time is 30 m³/Mu. The samples of each variety were taken on 23 March 2021, 2 April 2021, 13 April 2021, 19 April 2021 and 23 April 2021, and each variety had three replicates.

### Wheat point cloud data acquisition and data preprocessing

#### MVS-Pheno V2 plus platform and data acquisition.

**Figure 2.**
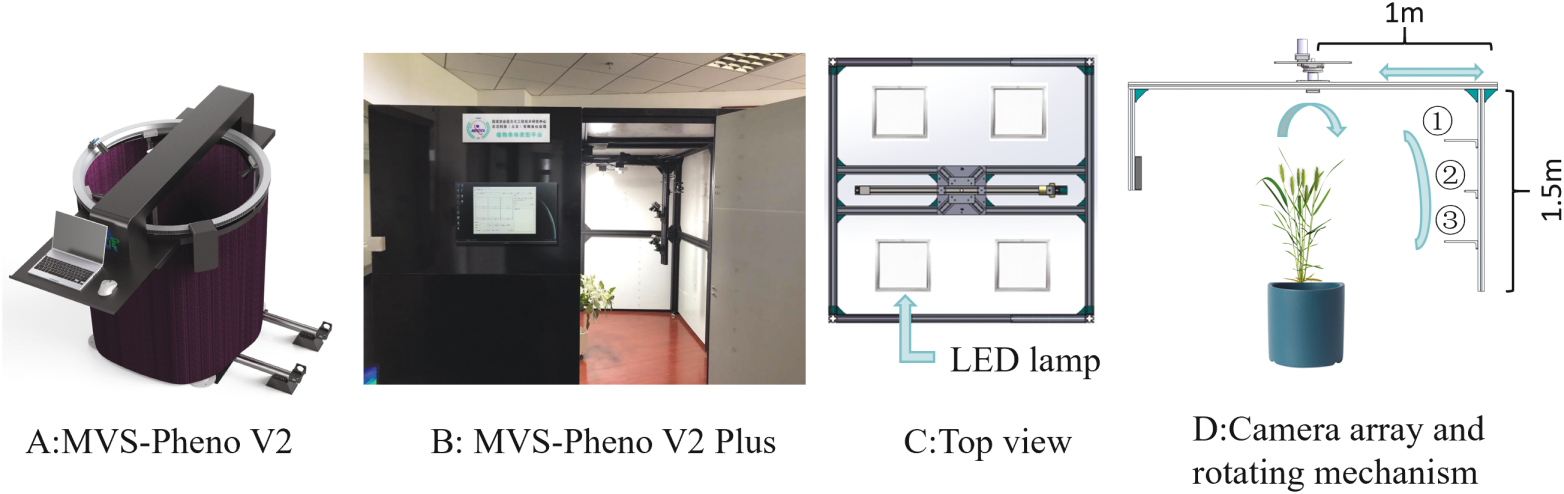
The improved multi-view image acquisition platform. (A) The overall appearance of MVS-Pheno V2. (B) Physical image of improved MVS-Pheno V2 Plus. (C) Top view of MVS-Pheno V2 Plus. (D) Internal camera array and rotation mechanism.

We used the MVS-Pheno V2 (Wu *et al.* 2022), a 3D phenotyping platform developed by our team, to complete the multi-view data collection for this experiment. MVS pheno V2 phenotypic platform is a multi-view image acquisition platform for single small plant, which can realize automatic and high-throughput data acquisition. To improve the quality of acquired multi-view images, we have made improvements based on this device version. The main improvements include three aspects: (i) replacing the original open-top structure with a closed box structure and installing four LED light sources on the top to improve the uniformity of the light sources (Fig. 2B and C); (ii) The collection angle range of the equipment is widened. After improvement, the maximum rotation radius of the rotating arm is 1 m, and the height of the rotating arm is 1.5 m. The rotation radius can be adjusted according to the width of the plant (Fig. 2D); (iii) multiple cameras are placed on the vertical rotating arm, and the height of the camera is adjustable. In addition, for cameras on different layers, the horizontal distance between the camera and the centre point of the device is adjustable. For general plants, the upper camera (Fig. 2D ①) and the lower camera (Fig. 2D ②) are closer to the plant, forming a spindle perspective structure.

For wheat plants planted in the field, we need to transplant them to the flowerpot for data acquisition. To prevent plants from wilting due to water shortage after transplanting, we need to properly water the plants to supplement water and complete data acquisition within half an hour after transplanting. When collecting data, we place the wheat plants to be photographed in the centre of the tray at the bottom of the device, install three cameras on the vertical rotating arm, set the camera acquisition interval to 2 s, set the rotation speed to 60 s/cycle, collect more than 90 images for each wheat plant sample. The overlap of two adjacent photos taken by each camera is more than 60%.

#### Multi-view 3D point cloud reconstruction and preprocessing.

We use algorithm technology solutions based on SFM and MVS to perform 3D reconstruction of multi-view images. We use Python language based on the SFM open source library openMVG and the MVS open source library openMVS to develop batch processing software for 3D point cloud reconstruction of plant multi-view images and automatically converting the obtained multi-view wheat images reconstruction 3D point cloud data. The point cloud generated based on the multi-view reconstruction technology not only includes the wheat plant point cloud but also the background point cloud. In addition, the coordinate system and point cloud scale of the point cloud generated by the multi-view reconstruction of different groups are also different, which will affect subsequent data processing. Therefore, we use a preprocessing algorithm to eliminate the background point cloud in the reconstructed point cloud and correct the coordinates to convert the coordinate values into real sizes. This part of the preprocessing mainly includes rough cropping, calibration plate extraction, scale correction, background point cloud removal and noise filtering. The detailed technical solution has been described in previous work and will not be repeated here(Wu *et al.* 2022). The reconstructed and corrected wheat plant point cloud data was used for the next step of model construction.

### Deep learning model for point cloud organ segmentation of wheat plant

#### Data set construction.

We constructed a data set including 100 wheat plant samples, of which 80 samples constituted the training set and the remaining 20 samples constituted the testing set. We used CloudCompare software to annotate the data, manually annotated the wheat plant point cloud into different instances of leaves, tillers and ears. Specifically, we use the segmentation tool in CloudCompare to segment each organ instance into different objects, and save each instance as a txt file that contains the coordinates and colour information of the point cloud. And organize the original point cloud files and organ instance point cloud files in the S3DIS dataset format (Armeni *et al.* 2016). During data annotation, some noisy point clouds will be manually removed. After that, we perform downsampling processing on all plant point clouds to keep the point cloud density of all samples relatively consistent.

#### Deep learning network model.

**Figure 3.**
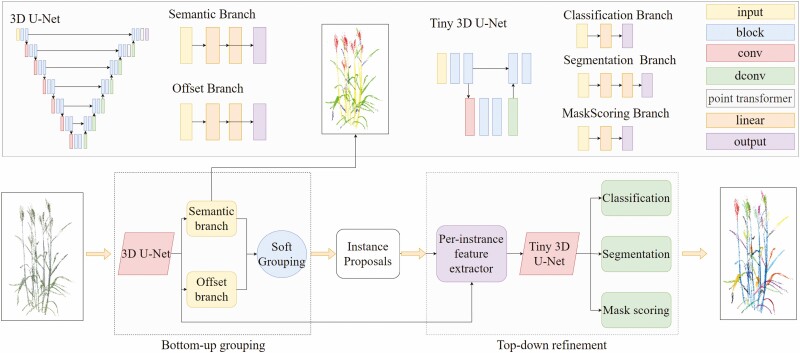
Structure of the improved SoftGroup model for segmentation. The model is divided into two parts: bottom-up grouping and top-down refinement. The detailed network structure of each part is supplemented at the top of the image.

In the past, point cloud instance segmentation for plant organs usually used networks such as PointNet (Qi *et al.* 2017a) and PointNet++ (Qi *et al.* 2017b) to segment the point cloud semantically and clustered semantic segmentation results to achieve instance segmentation. However, the leaves of wheat plants are dense, and the cross phenomenon is common. The traditional method of semantic segmentation and clustering cannot achieve the segmentation of wheat leaf instances. Therefore, in this paper, we trained on the constructed wheat point cloud dataset based on SoftGroup (Vu *et al.* 2022) point cloud instance segmentation network, aiming to achieve instance segmentation of wheat leaves, tillers and wheat ears. SoftGroup is a network model used to handle the task of point cloud instance segmentation, and it can realize semantic segmentation and instance segmentation of point cloud at the same time. The overall architecture of the network is shown in Fig. 3, which is divided into two stages: bottom-up grouping and top-down refinement.

In the bottom-up grouping stage, the network first takes the wheat point cloud as the input, and each point contains coordinate and colour information. These points are voxelized and transformed into ordered voxel grids for subsequent convolution operations. Then, these voxel data are extracted through a 3D U-Net backbone network using submanifold sparse convolution (Graham *et al.* 2018) and then restored from the voxel data back to the point cloud to extract features for each point. In addition, here we have made some changes to the 3D U-Net backbone network, adding the point transformer module (Zhao *et al.* 2021) in the first and second layers, which will pay more attention to the local neighbourhood information of each point and encode the location information to better extract the characteristics of the point cloud. After that, the network is divided into two branches: the semantic segmentation branch and the offset prediction branch. The semantic branch is composed of two layers of multilayer perceptron (MLP), which outputs the semantic score of each point. The offset branch is also composed of two layers of MLP, which outputs the offset vector of each point to the centroid of its instance, the offset vectors help to make the points of the same instance more centralized. In this step, the network model can directly output the semantic segmentation results of the wheat plant point cloud. Finally, the grouping module takes semantic scores and offset vectors as inputs and generates instance proposals. The semantic classification of each point is determined by the score threshold *τ*, if the semantic scores of multiple categories at the point are greater than *τ*, the point is temporarily associated with multiple categories to reduce the impact of semantic segmentation errors on instance segmentation. Subtract each point from its offset vector, moving the point toward the centre of the instance. Then, use ball query to find the neighbouring points of each point for point clouds belonging to the same class, and then use breadth-first search (BFS) to generate a set of proposals, each proposal representing a possible object instance.

The top-down refinement stage classifies and refines the instance proposals in the bottom-up grouping stage. First, the proposal, point coordinates and point features are fused and voxelized, and then the voxelized features are input into a lightweight 3D u-net network for processing. The processed feature data is sent to three branches: the classification branch, the segmentation branch and the mask scoring branch. The classification branch is a full connection layer used to determine the category of the instance. The segmentation branch is a two-tier MLP, which is used to determine whether a point belongs to this instance. The mask score branch is a full connection layer that estimates the IoU score of the predicted instance. Finally, the mask score and classification score are multiplied to obtain the final confidence score, and the results of wheat point cloud organ instance segmentation are output.

#### Loss function.

The SoftGroup network can be trained in an end-to-end manner using multitask loss. The loss function includes the loss of semantic and offset in the bottom-up grouping stage and the loss of classification, segmentation, and mask scores in the top-down refinement stage.

Cross entropy loss and L1 regression loss are used to train semantic branches and offset branches, respectively.


$$\begin{array}{l} \begin{array}{l} L_{semantic}=\frac{1}{N}\underset{i=1}{\overset{N}{\sum }}\;CE\left(s_{i},s_{i}^{*}\right)\; \end{array} \end{array}$$ (1)

$$\begin{array}{l} \begin{array}{l} L_{of\;fset}=\frac{1}{\overset{N}{\underset{i=1}{\sum }}1\left\{p_{i}\right\}}\overset{N}{\underset{i=1}{\sum }}1\left\{p_{i}\right\}{∥o_{i}-o_{i}^{*}∥}_{1}\; \end{array} \end{array}$$ (2)

where *N* is the number of point clouds, $s_{\;}^{*}$ is a semantic tag, $o_{\;}^{*}$ is the offset label, and $\;1\left\{p_{i}\right\}$ is the indicating function, indicating whether the point $p_{i}$ belongs to any instance.

Cross entropy loss, binary cross-entropy loss and L2 regression loss are used for the classification branch, the segmentation branch and the mask scoring branch, respectively.


$$\begin{array}{l} \begin{array}{l} L_{class}=\frac{1}{K}\underset{k=1}{\overset{K}{\sum }}\;CE\left(c_{k},c_{k}^{*}\right)\; \end{array} \end{array}$$ (3)

$$\begin{array}{l} \begin{array}{l} L_{mask}=\frac{1}{\overset{K}{\underset{k=1}{\sum }}1\left\{m_{k}\right\}}\overset{K}{\underset{k=1}{\sum }}1\left\{m_{k}\right\}BCE\left(m_{k},m_{k}^{*}\right)\; \end{array} \end{array}$$ (4)

$$\begin{array}{l} \begin{array}{l} L_{mask_{score}}=\frac{1}{\overset{K}{\underset{k=1}{\sum }}1\left\{r_{k}\right\}}\overset{K}{\underset{k=1}{\sum }}1\left\{r_{k}\right\}{∥r_{k}-r_{k}^{*}∥}_{2}\; \end{array} \end{array}$$ (5)

where *K* is the total number of proposals, $c_{\;}^{*}$, $m_{\;}^{*}$ and $r_{\;}^{*}$ are classification, segmentation and mask scoring objectives, respectively. 1{.} indicates whether the proposal is a positive sample.

The total loss of the network is:


$$\begin{array}{l} \begin{array}{l} L=L_{semantic}+L_{of\;fset}+L_{class}+L_{mask}+L_{mask_{score}}\; \end{array} \end{array}$$ (6)

#### Training hyperparameters.

The network model we used is based on the PyTorch deep learning framework. We used the Adam optimizer to perform training, with a total of 1000 epochs. The batch size of the model is set to 4, and four repetitions of learning are performed for each sample to strengthen the training effect. The initial value of the learning rate is set to 0.001, and it is dynamically scheduled by a cosine annealing. The voxel size is set to 0.1, the score threshold *τ* of semantic score in grouping is 0.2, and the search radius of the nearest neighbour is 0.3. In the training phase, we use the HAIS training model as the pre-training basis for the backbone network to further train the backbone network. After the backbone network training, we freeze the parameters of the backbone network and train other parts of the network. The above experimental results were completed on a computer using Intel Core i7-12700, 16 GB RAM and NVIDIA RTX 3070TI GPU, running Ubuntu 20.04 operating system.

### 3D Structure reconstruction of wheat plant

#### 3D structure reconstruction of wheat tiller.

When collecting point cloud data, the 3D reconstruction point cloud of wheat tiller may appear sparse, noisy or missing due to factors such as shooting angle and leaf occlusion, which affects the accuracy of tiller segmentation and 3D reconstruction. Therefore, in this part, we repair the point cloud of the tiller based on the semantic segmentation results of the tiller to make it more regular. Then we use a top-down search method to segment the tiller and reconstruct its 3D structure.

**Figure 4.**
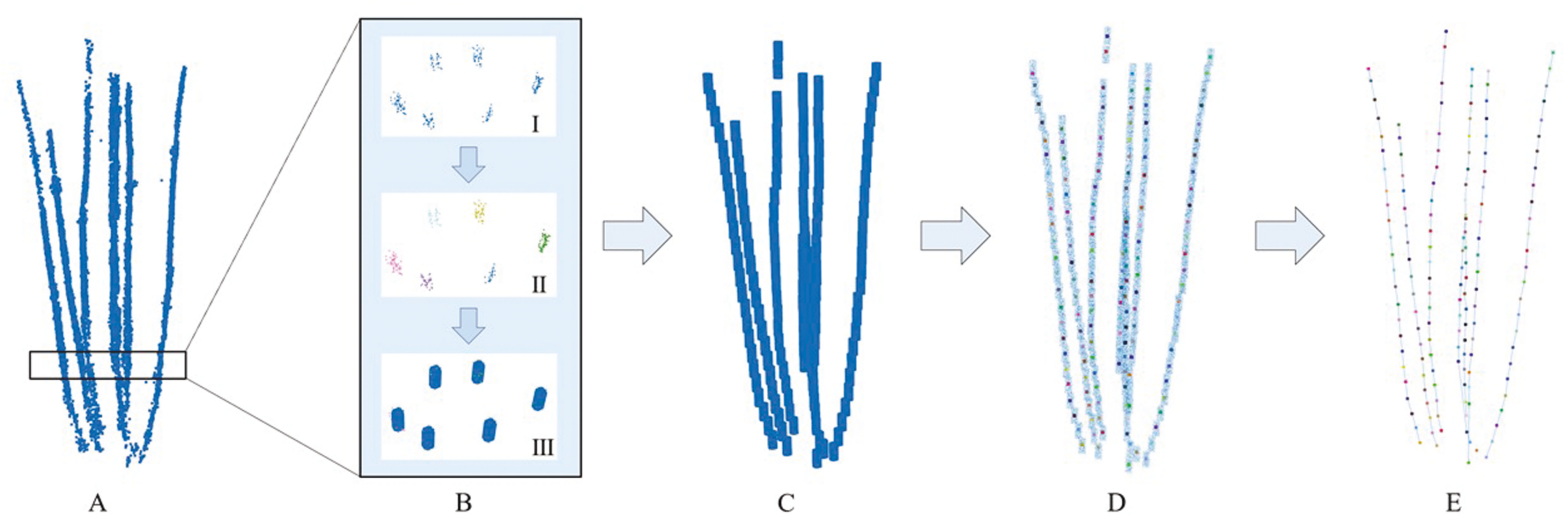
Schematic diagram of wheat plant tiller repair and skeleton extraction. (A) Schematic diagram of segmenting tiller point cloud. (B) Schematic diagram of tiller point cloud repair. Use the DBSCAN clustering algorithm on the segmented point clouds and replace each cluster with a cylindrical point cloud. (C) Results of tiller point cloud repair. (D) Extract the centroid point of each cluster. (E) 3D reconstruction results of tiller skeleton.

First, we extract the point cloud of the tiller from the semantic segmentation and segment it every 2 cm from the lowest end of the tiller. Each segment is marked as $SP_{n}$, which n is the sequence of segments from bottom to top, and the segmentation results are shown in Fig. 4A. Next, we use the DBSCAN clustering algorithm to cluster the segmented point clouds, and the clustering results are shown in Fig. 4B. Then, we get the central points $SP_{n}^{m}$ of each cluster, which m is the number of each cluster, and generate a cylindrical point cloud with a height of 2 cm and a radius of 0.5 cm at the centre of the cluster to replace the original tiller point cloud, as shown in Fig. 4B. Finally, the overall repaired tiller point cloud is shown in Fig 4C.

It is worth noting that there will be some limitations if the clustering algorithm is directly used to segment the repaired tiller point cloud. For example, two intersecting tillers are incorrectly classified as one instance, or a tiller is wrongly divided into two tillers due to semantic segmentation errors, serious loss of some point clouds, and other reasons. To solve the problems, we propose a search algorithm to extract accurate tiller instances and their 3D skeleton structure.

We propose a top-down search method for tiller instance segmentation and skeleton extraction. First, input as the centre points ${SP}_{n}^{m}$ of each cluster, take points ${SP}_{n}^{m}$ as the skeleton points of tiller stalk, the number of skeleton points is recorded as $SP_{num}$, and then the skeleton points ${SP}_{n}^{m}$ are sorted according to the height of the points. Then calculate the horizontal distance $h_{i,j}$ and vertical distance $v_{i,j}$ between each skeleton points, in which i and j is two points in the skeleton points ${SP}_{n}^{m}$, i ranges from 1 to $SP_{num}$, j ranges from i to $SP_{num}$. Then, multiply the horizontal distance $h_{i,j}$ and vertical distance $v_{i,j}$ by the weight α and β and then added together to obtain the score table $score_{i,j}$, the calculation formula of score is as follows:


$$\begin{array}{l} score_{i,j}=\;\alpha \;\times \;h_{i,j}+\;\beta \;\times \;v_{i,j} \end{array}$$

This score table represents the score between point i and point j, the lower the score, the closer the distance between point i and point j, and the greater the probability that points i and point j belong to the same tiller. After establishing the score table, we search from top to bottom according to the height of the points ${SP}_{n}^{m}$, and add the points belonging to the same tiller to the point set $S_{t,k}$, where t is the serial number of the tiller and k is the serial number of the points in the set. In addition, combined with the morphological characteristics of wheat, we optimized the search process by limiting the slope and distance between two lines to ensure the accuracy of the search effect. On this basis, we filter the obtained point set and exclude the set with less than 4 points to avoid the interference caused by noise and classification errors. Finally, connect the points in the same set in turn to get the skeleton of the tiller, as shown in Fig. 4E.

It should be noted that the 3D reconstructed point cloud is partially incomplete at the bottom, which will affect subsequent parameter extraction. In order to correct this error, we extend the bottom of each tiller downward from the original height $H_{S_{t,0}}$ to the lowest point $H_{0}$ of all tillering points of the wheat plant to reduce the error.

#### 3D structure reconstruction of leaf.

**Figure 5.**
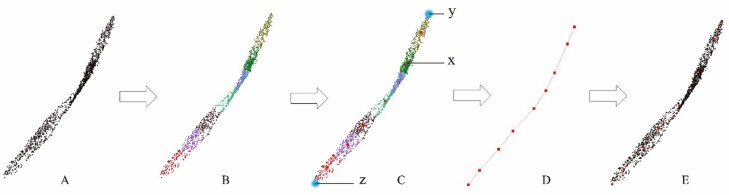
Schematic diagram of wheat plant leaf skeleton extraction. (A) Leaf original point cloud. (B) K-means clustering results. (C) Schematic diagram of clustering segment centre point extraction and leaf endpoint extraction. (D) 3D reconstruction results of leaf skeleton (E) Skeleton and leaf fitting.

This section mainly describes how to reconstruct the 3D structure of the leaves of wheat plants. First, for each leaf point cloud instance, we use the k-means clustering algorithm to evenly divide it into multiple point cloud segments $L_{i}$ along the direction of the leaf length, where i is the sequence number of the point cloud segment. The number of clustering clusters is determined according to the size (the number of points of the leaf) of the leaf. Based on real machine testing experience, the threshold we set is: when the number of point clouds of the leaf instance is less than 1000, the number of clusters is set to 4; when the number of point clouds is greater than 1000, the number of clusters is set to 7, and the clustering results are shown in Fig. 5B. For each segmented point cloud segment $L_{i}$, we calculate the centroid of its point cloud $LP_{i}$ as the reference point on the leaf skeleton, the result is shown as the red point in Fig. 5 C.

For the extraction of the two endpoints of the leaf, as shown in Fig. 5C, we select the centroid x of the point cloud segment $L_{1}$ adjacent to the edge point cloud segment $L_{0}$, and calculate the point farthest from x in the $L_{0}$ point cloud segment y, let point y be regarded as an endpoint. Process the other endpoint of the leaf in the same way to obtain the other endpoint z of the leaf. Finally, calculate the distance between endpoints z and z and all centroids $LP_{i}$ and connect the two points with the smallest distance each time according to the spacing distance to approximate the skeleton of the leaf. The result is shown in Fig. 5D, and the fitting situation of the leaf skeleton and the leaf point cloud is shown in Fig. 5E.

#### Location of leaf attachment point.

The attachment point of leaf is the connection between leaf and tiller, usually located at the base of leaf. In the previous section, we extracted the two endpoints y and z of the leaf. However, these two endpoints cannot directly determine which is the base and which is the tip of the leaf. To solve this problem, we considered the relationship between the endpoint position and the whole wheat point cloud to determine the base and tip of the leaf. In general, the leaves at the bottom of the wheat plant will be slightly bent and spread around, and the end point of the leaf base is closer to the centre of the whole point cloud of the wheat plant. The leaves at the top of the wheat plant, especially those near the ear, tend to grow vertically or slightly obliquely upward, and the height of the end point at the base of the leaf is lower than that at the tip. According to the morphological characteristics of wheat, the leaf base point in the skeleton can be extracted, that is, the attachment points of wheat leaves.

However, due to the complexity of wheat plant growth, the above extraction method of leaf base points may produce errors. For example, the leaves at the bottom of the plant do not completely spread around, or some leaves are bent, which will affect the extraction results. Therefore, we need to correct the results. The calibration steps are as follows: first, we compare the distance between the leaf base point and all skeleton points ${SP}_{n}^{m}$ in Section 3D structure reconstruction of wheat tiller, select the nearest skeleton points and record the minimum distance as minD. Next, calculate the average distance from the base of all leaves to the nearest tiller skeleton points, and record it as avgD. Then, compare the minD and avgD of each leaf in turn. If the minD is greater than 1.75 times (values based on experience) the avgD, we think that the base point may be extracted incorrectly. At this time, calculate the distance from the other end point of the leaf to its nearest tillering skeleton points $minD_{2}$. At this time, calculate the distance $minD_{2}$ from the other endpoint of the leaf to its nearest tillering skeleton points. If $minD_{2}$ is less than minD, it is considered that the base point extracted for the first time is wrong, and take the other endpoint as the correct leaf base point. If $minD_{2}$ is greater than minD, it is considered that the base point extracted for the first time is correct, and the original result is retained. Finally, according to the modified leaf base point, we reselected the nearest skeleton points as the leaf attachment point, and took the tiller of the skeleton points as the attachment tiller.

#### 3D reconstruction of wheat plants.

In Sections 3D structure reconstruction of wheat tiller and 3D structure reconstruction of leaf, we successfully extracted the tiller skeleton of wheat plant and the leaf skeleton. In Section Location of leaf attachment point, we calculated the position information of the leaf attachment point and the tiller attached to the leaf. Based on the above information, we can accurately assemble the leaves to the corresponding tillers and correct positions, and then complete the 3D phytomer assembly and reconstruction of wheat plants based on data-driven.

### Extraction of plant morphological parameters

In this paper, 18 wheat plant morphological-related parameters were obtained by using 3D point cloud and 3D reconstruction results. These parameters can be divided into plant morphological structure parameters and tiller internode parameters. The specific parameters are shown in Table 1.

**Table 1.** Overview of wheat plant morphological parameters extraction based on 3D reconstruction.

Parameter category	Parameter name	Unit	Description
Plant morphological structure	Number of tillers		The total number of tillers
	Main stem		Find the main stem in the tillers
	Tiller length	cm	The length of tillers from the base to the top
	Angle between tiller and main stem	°	The angle between tillering and main stem
	Tiller azimuth	°	The angle between the tiller and the *X*-axis when projected onto the horizontal plane xOy
	Plant height	cm	Vertical distance from the ground to the highest point of the plant
	Girth	cm	The diameter of the polygonal circumcircle formed by the tillers at 1/2, it reflects the looseness of wheat plant tillers
Tiller internode	Number of leaves		Total number of leaves
	Leaf length	cm	The length of the leaf from the base to the tip
	Leaf width	cm	The distance of the widest part of the leaf
	Leaf angle	°	The angle between the leaf and the attached tiller
	Leaf azimuth	°	The angle between the leaf and the *X*-axis when projected onto the horizontal plane xOy
	Leaf attachment point	(cm, cm, cm)	3D coordinates of the birth position of leaves on tillers
	Leaf venule	((*x*_1_, *y*_1_, *z*_1_), …, (*x*_*n*_, *y*_*n*_, *z*_*n*_))	Key nodes on leaf skeleton
	Number of ears		The total number of ears
	Ear length	cm	The length of an ear from the base to the top
	Ear attachment point	(cm, cm, cm)	The 3D coordinates of the position of the ear attached to the tiller
	Ear volume	cm³	Volume of ears

#### Parameterized calculation of plant morphological structure.

The number of tillers was obtained from the 3D reconstruction results of tillers in Section 3D structure reconstruction of wheat tiller. For the selection of the main stem, we choose the tiller located at the centre of the plant and with a longer length as the main stem. To calculate the tiller length, we use the tiller point set $S_{t,k}$ in Section 3D structure reconstruction of wheat tiller and accumulate the sum of the distance between the centre points belonging to the same tiller. The calculation formula is as follows, where dist (*x*, *y*) represents the distance between *X* and *Y* points:


$$\begin{array}{l} \begin{array}{l} H_{t}=\underset{n=0}{\overset{k-1}{\sum }}\;dist\left(S_{t,n},S_{t,n+1}\right)+\left(H_{S_{t,0}}-\;H_{0}\right)\; \end{array} \end{array}$$ (7)

For the calculation of tiller angle parameters, the skeleton points at 1/3 height and 2/3 height of tiller and main stem are respectively taken to connect them to form the tiller vector and main stem vector. The included angle $\alpha_{s}$ between the two vectors is calculated to obtain the included angle between tiller and main stem. The tiller azimuth $\beta_{s}$ is obtained by calculating the included angle between the projection of tiller vector on the horizontal plane xOy and the positive direction of the *x*-axis.

The plant height of wheat is obtained by calculating the height difference between the highest point and the lowest point in the wheat point cloud. The wheat girth is defined as the diameter of the circumscribed circle of the convex hull composed of tillers at 1/2 height of wheat. Firstly, the skeleton points at 1/2 height of each tiller of wheat are extracted to calculate the convex hull, and then Welzl algorithm is used to calculate the circumscribed circle centre and diameter of the convex hull, and the diameter is used as the circumference parameter of wheat.

#### Parameterized calculation of tiller internode.

The number of leaves is directly obtained from the segmentation results of wheat point cloud instances in Section Deep learning model for point cloud organ segmentation of wheat plant. To calculate the leaf length, we use the $LP_{i}$ of leaf skeleton points and endpoints A and C extracted in Section 3D structure reconstruction of leaf. The distance between two adjacent points on the leaf skeleton curve is small and relatively uniform, and the curvature of the leaf skeleton does not change significantly. Therefore, the length of the leaf can be obtained by summing the distances of all adjacent points on the leaf skeleton. The calculation formula is as follows, where dist (X, Y) represents the distance between X and Y points:


$$\begin{array}{l} \begin{array}{l} L=\underset{n=0}{\overset{i-1}{\sum }}\;dist\left(LP_{n},LP_{n+1}\right)+dist\left(LP_{0},y\right)+\;dist\left(LP_{i},z\right)\; \end{array} \end{array}$$ (8)

As for the calculation of leaf width, we select two point cloud segments $LP_{\left\lfloor i/2\right\rfloor }$ and $LP_{\left\lfloor i/2\right\rfloor +1}$, which are in the middle of the leaf, use the principal component analysis (PCA) algorithm to determine the length, width and thickness of the leaf point cloud, and then calculate the projection of the point cloud segments $LP_{\left\lfloor i/2\right\rfloor }$ and $LP_{\left\lfloor i/2\right\rfloor +1}$ in the width direction, and calculate the projected length as the leaf width.

For the calculation of leaf angle parameters, the angle between tiller and leaf is obtained by calculating the angle $\alpha_{l}$ between the growth direction of leaf base and the local growth direction of tillers. The leaf azimuth is obtained by calculating the angle $\beta_{l}$ between the projection of the vector from the leaf base to the highest point of the leaf on the horizontal plane xOy and the positive direction of the *x*-axis. The leaf attachment point and skeleton point have been described in sections 3D structure reconstruction of leaf and Location of leaf attachment point.

The number of ears can be directly obtained from the segmentation results of wheat point cloud instances in Section Deep learning model for point cloud organ segmentation of wheat plant. The length of ear can be calculated by using PCA algorithm to obtain the vector of ear length direction, and then projecting the ear point cloud to this direction to calculate the length parameter of ear. The ear volume is calculated based on convex hull. First, the convex hull of the ear is calculated, and then the overall volume of the ear is calculated by calculating the directed volume of all faces of the convex hull. The point with the lowest ear height was selected as the point of attachment for the parameter of ear attachment point.

### Evaluation indicators

We evaluate the segmentation results of wheat plants’ point cloud in the test set using quantitative indicators. We select accuracy (ACC) and intersection union ratio (IoU) as the main indicators to evaluate the semantic segmentation accuracy of the wheat point cloud. Accuracy refers to the ratio between the number of correctly classified point clouds and the total number of point clouds in the segmentation result. IoU refers to the ratio of intersection and union between predicted segmentation and true segmentation. Specifically, the true positives (TP) represent the number of points correctly identified as the current semantic category, the true negatives (TN) represent the number of points correctly identified as non-current semantic categories, the false positives (FP) represent the number of points incorrectly identified as the current semantic category, and the false negatives (FN) represent the number of points not correctly identified as the current semantic category. The calculation formula of acc and IoU is as follows:


$$\begin{array}{l} \begin{array}{l} ACC=\frac{TP+TN}{TP+TN+FP+FN}\; \end{array} \end{array}$$ (9)

$$\begin{array}{l} \begin{array}{l} IoU=\frac{TP}{TP+FP+FN}\; \end{array} \end{array}$$ (10)

In the evaluation of instance segmentation, we use average precision (AP) and average recall (AR) as evaluation criteria. Among them, AP_50_ and AP_25_ refer to the score under the IoU threshold of 50% and 25%, the comprehensive AP is the average score when the IoU threshold increases from 50% to 95% in steps of 5%, RC_50_ and RC_25_ refer to the recall rate under the IoU threshold of 50% and 25%.

To evaluate the accuracy of the proposed method for extracting plant morphological parameters based on segmentation results, we compared the plant morphological parameters extracted from segmentation results with the actual measured values. In this evaluation process, we use the correlation coefficient ($R^{2}$) and root mean square error (RMSE) to quantify the accuracy of the analysis. The specific calculation formula is as follows:


$$\begin{array}{l} \begin{array}{l} R^{2}=1-\frac{\overset{n}{\underset{i=1}{\sum }}{\left(x_{i}-{\hat{x}}_{i}\right)}^{2}}{\overset{n}{\underset{i=1}{\sum }}{\left(x_{i}-{\bar{x}}_{i}\right)}^{2}}\; \end{array} \end{array}$$ (11)

$$\begin{array}{l} \begin{array}{l} RMSE=\;\sqrt{\frac{1}{m}\Sigma_{i=1}^{n}{\left(x_{i}-\hat{x}i\right)}^{2}}\; \end{array} \end{array}$$ (12)

where n represents the total number of samples to be evaluated, and $x_{i}$ represents the value actually measured manually, ${\hat{x}}_{i}$ represents the plant morphological parameter value extracted by the algorithm, ${\bar{x}}_{i}$ represents the average of all manual measurements.

## Results

### Quantitatively evaluation of segmentation results

**Figure 6.**
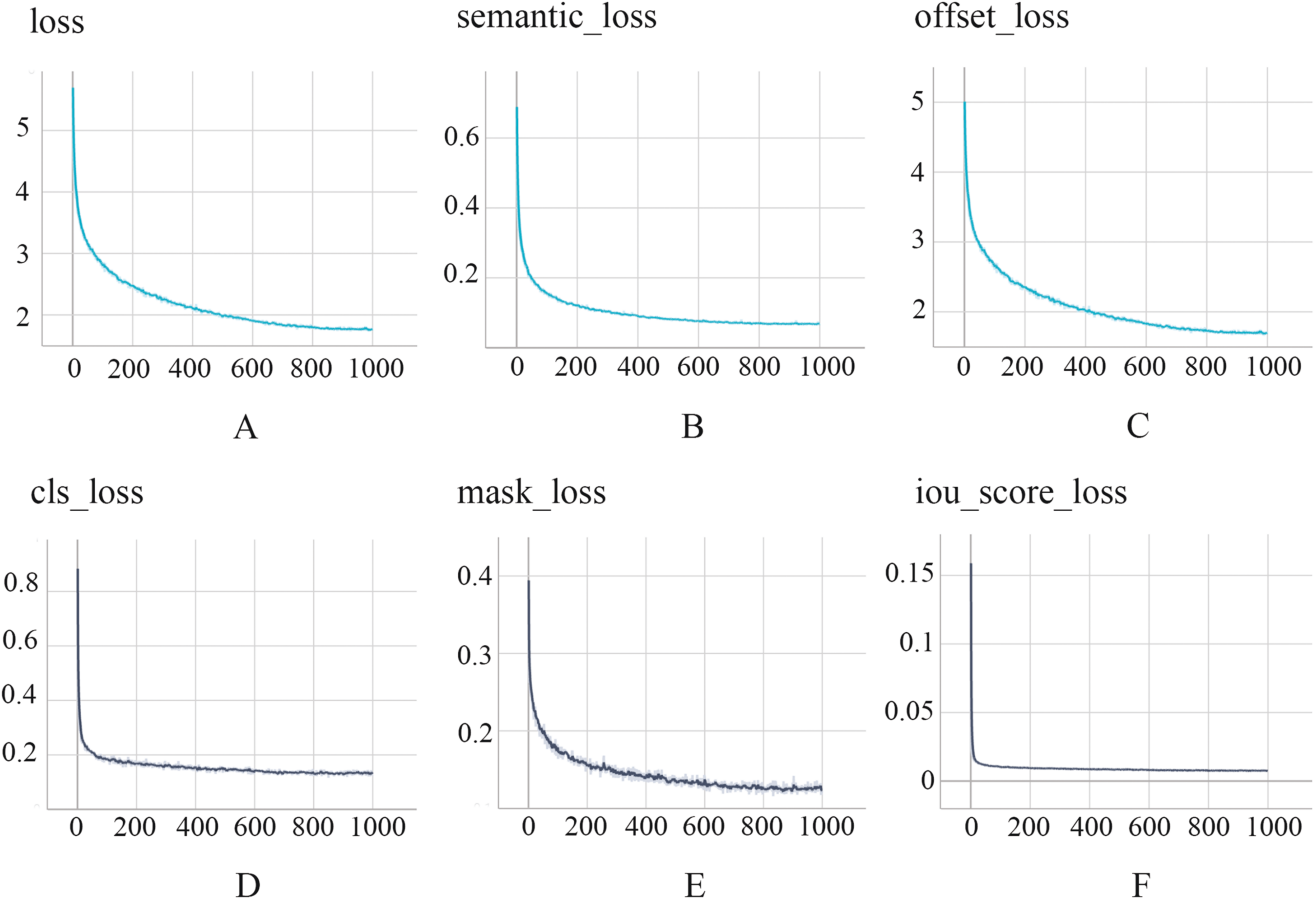
Training losses of the segmentation model. (A) Changes in semantic segmentation loss during backbone network training. (B) Changes in offset loss during backbone network training. (C) Sum of semantic segmentation loss and offset loss. (D) Changes in classification loss during non-backbone network training. (E) Changes in mask score loss during non-backbone network training. (F) Changes in IoU score loss during non-backbone network training.

In the segmentation evaluation part, we first analysed the results of model learning by observing the change of loss function value during the training process. The change in the loss value in the fine-tuning training phase of the backbone network is shown in Fig. 6A–C. It can be observed that the training loss decreases rapidly within the first 600 epochs and then enters a stable state. Finally, the semantic segmentation loss value decreases to 0.067, the offset loss value decreases to 1.694, and the comprehensive loss value is 1.762. The stage loss value of the non-backbone network of the training model changes is shown in Figure 6D–F. The training loss shows a rapid downward trend in the first 400 epochs and then gradually tends to be stable. Finally, the classification loss value decreased to 0.129, the mask score loss value decreased to 0.122, the IoU score loss value decreased to 0.007, and the total loss value was 2.516.

The following is to evaluate the results of wheat plant segmentation in the test set through quantitative indicators. In terms of semantic segmentation accuracy, Our model achieves 95.1% accuracy and 84.0% mean IoU (mIoU) on the wheat point cloud test set. For each organ category, the IoU of leaves, tillers, and ears were 94.2%, 80.7% and 77.1%, respectively. In terms of instance segmentation accuracy, the specific indicators of the proposed model for instance segmentation performance of different organs are shown in Table 2.

**Table 2.** Accuracy evaluation of the instance segmentation result in the testing set.

	AP	AP50%	AP25%	RC50%	RC25%
Leaf	0.677	0.677	0.782	0.861	0.798
Tiller	0.238	0.457	0.697	0.505	0.774
Wheatear	0.711	0.845	0.887	0.852	0.889
Average	0.542	0.695	0.815	0.718	0.843

### Visualization of segmentation and reconstruction results

**Figure 7.**
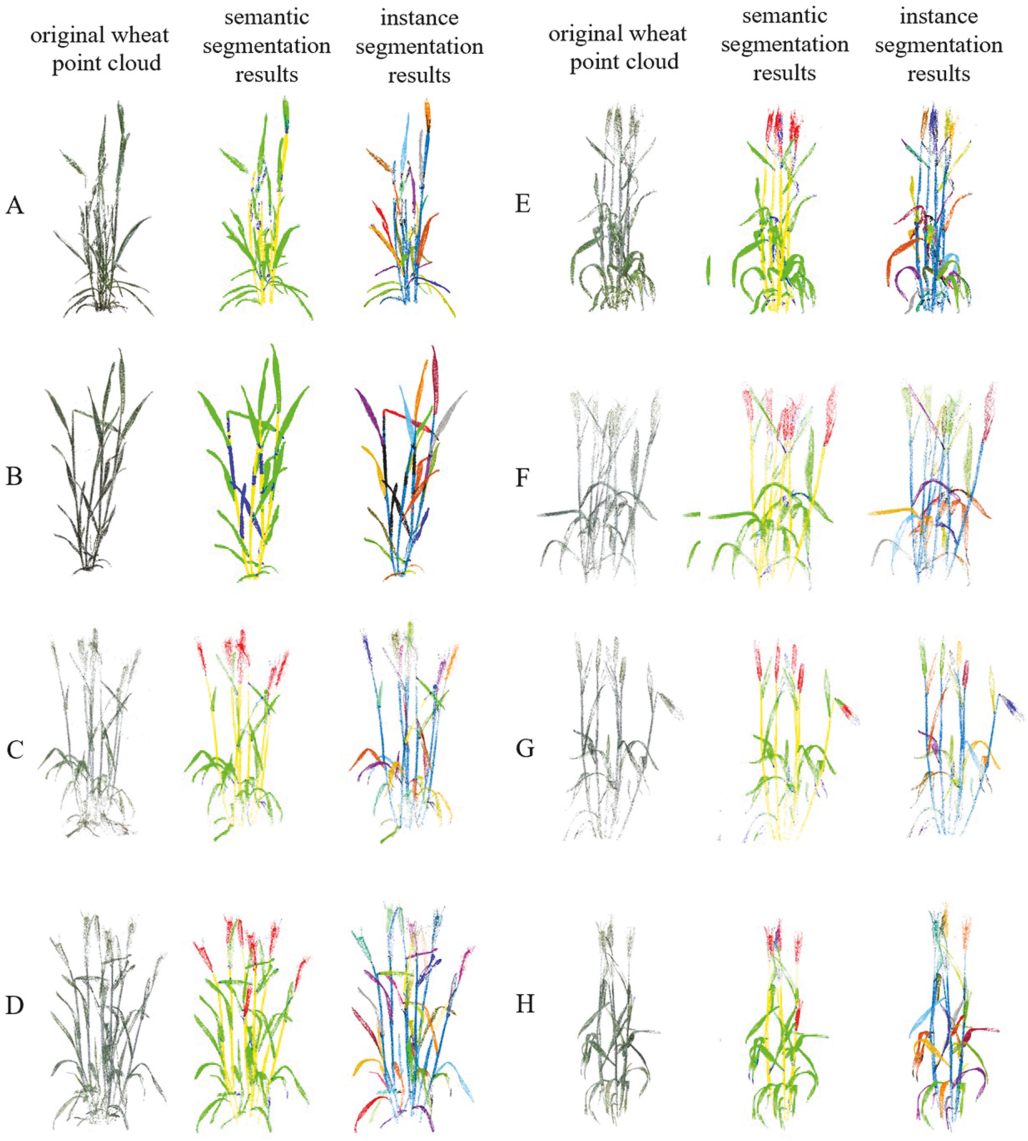
Visualization of wheat point cloud segmentation results. Alphabet numbering represents different varieties of wheat plants. The first column is the processed original wheat point cloud. The second column is the semantic segmentation results. The third column is the instance segmentation results.

In this part, we visually analysed the results of point cloud segmentation and wheat 3D reconstruction. The result of point cloud segmentation is shown in Fig. 7. The first column presents the raw point cloud data of the pretreated wheat plants. The second column presents the semantic segmentation results of the point cloud: green represents the leaf, yellow represents the tiller, red represents the wheat ear and blue represents the unrecognized part. The third column shows the result of instance segmentation, the instance segmentation result of the tiller is replaced by the semantic segmentation result of the tiller and all the point clouds of the tiller are displayed in dark blue. The point clouds of leaves and ears of wheat are subdivided into independent instances, and each instance is distinguished by different colours.

**Figure 8.**
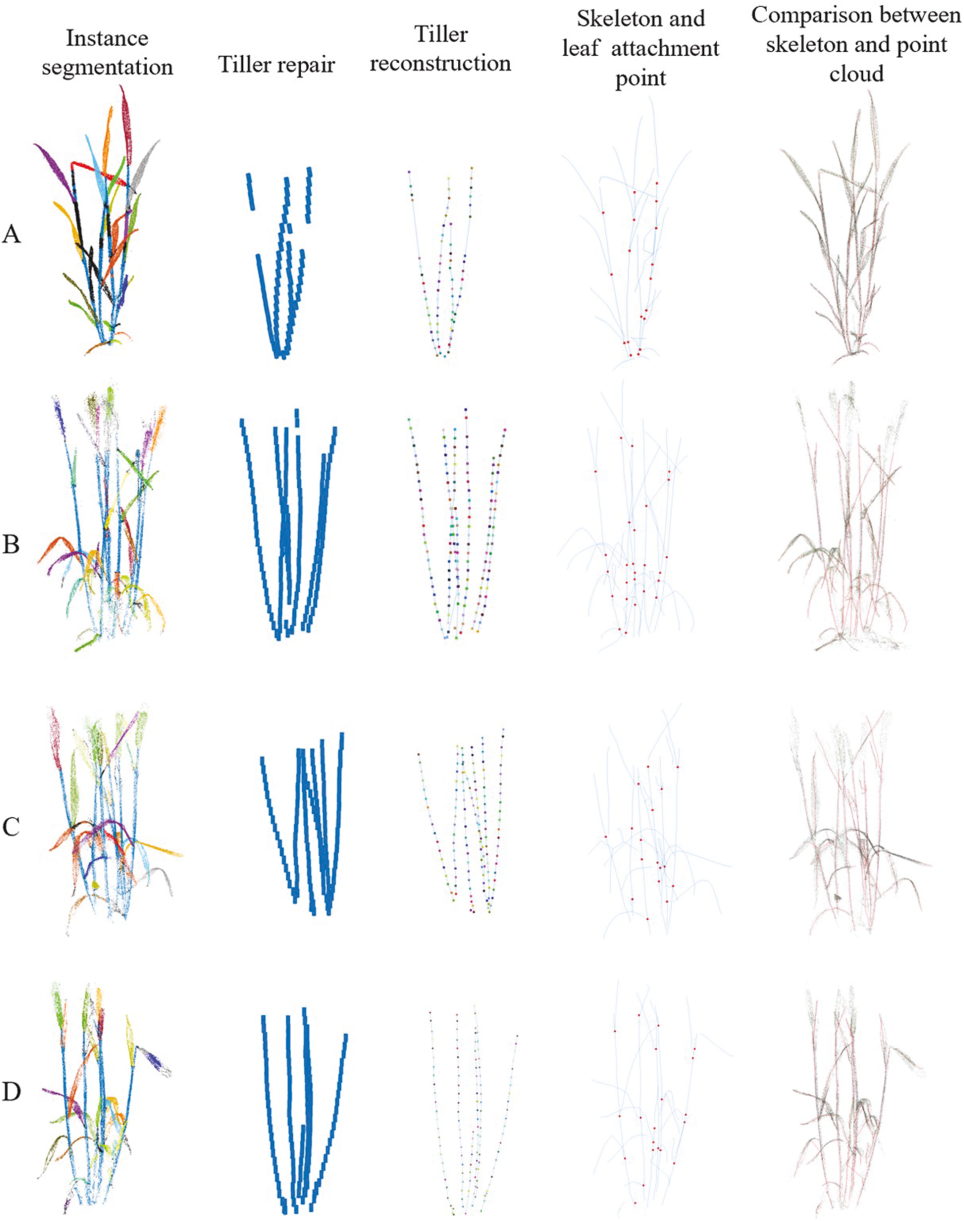
Visualization of wheat 3D reconstruction results. Alphabet numbering represents different varieties of wheat plants. The first column is the point cloud instance segmentation results. The second column is the tiller repair results. The third column is the tiller reconstruction results. The fourth column is the tiller and leaf reconstruction results and the attachment point extraction results. The fifth column is the comparison of the fitting degree between the skeleton and the original point cloud.

The results of 3D reconstruction are shown in Fig. 8. The first column shows the segmentation results of the wheat point cloud instance. The second column shows the effect of the wheat tiller completion. The third column presents the extraction results of the wheat tiller skeleton. The fourth column shows the skeleton of wheat leaves and tillers, and the attachment point of the leaf on the tillers, which is marked with red dots. Finally, the fifth column is the comparison between the results of wheat leaves and tiller skeleton extraction and the original point cloud, we overlay the extracted skeleton with the original point cloud to reflect the degree of fit between the extracted leaf and tiller skeleton and the original point cloud, where the skeleton is a red line.

By observing the semantic and instance segmentation results of wheat tillers, leaves, and ears, we observed that the output predicted by the model was highly consistent with the real situation. This similarity shows that the model has achieved good performance in the task of wheat point cloud instance segmentation and can accurately distinguish tillers, leaves and ears.

For the task of tiller repair and skeleton extraction, the results show that the repaired tiller point cloud is more regular and continuous than the original data. Based on the skeleton information extracted from these repaired point clouds, the geometric shape of the skeleton highly overlaps with the original tiller point cloud.

For the extraction of the leaf skeleton and the prediction of the position of the leaf attachment point, the predicted leaf skeleton in the result graph has a high degree of coincidence with the real leaf point cloud, and the prediction of the position of the attachment point is also highly consistent with the actual observation, is also shows the accuracy of this algorithm.

### Evaluation of extracted plant morphological parameters

**Figure 9.**
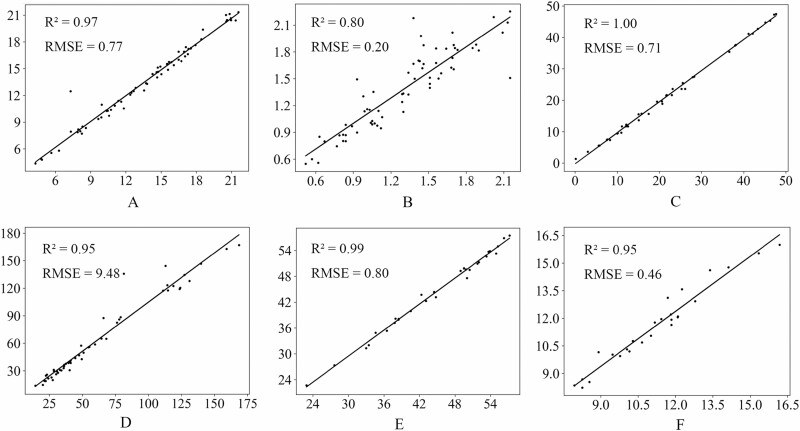
Comparison of some plant morphological parameters obtained by the algorithm and actual values. (A) Leaf length. (B) Leaf width. (C) Leaf insertion point height. (D) The angle between tillers and leaves. (E) Tiller length. (F) Ear length.

Fig. 9A and b shows the extraction results of leaf length and leaf width respectively. The correlation coefficient ($R^{2}$) of the leaf length parameter was 0.97, and the root mean square error (RMSE) was 0.77, indicating that the difference between the predicted value and the actual value was small. For the leaf width parameter, *R*^2^ is 0.80, RMSE is 0.20, and the correlation is low. Fig. 9C shows the extraction results of the height of leaf implantation sites. *R*^2^ reaches 1.00 and RMSE is 0.71, indicating that the extraction results of leaf implantation sites are highly correlated with the actual value. In addition, the average deviation between the predicted value and the actual value of leaf implantation sites in the horizontal direction is 1.44 cm, which proves that this method can identify and locate the growth points of wheat leaves with high accuracy in 3D space. Fig. 9D shows the extraction results of stem leaf angle, with *R*^2^ of 0.95 and RMSE of 9.48, indicating that the extraction results of stem leaf angle are highly correlated with the actual value. The extraction results of tiller length parameters are shown in Fig. 9E, with *R*^2^ of 0.99 and RMSE of 0.80, showing that the prediction of tiller length by this method is highly consistent with the actual measured value. The extraction result of ear length is shown in Fig. 9F, with *R*^2^ of 0.95 and RMSE of 0.46, indicating that the extraction result is highly correlated with the actual value.

From the above analysis, it can be seen that the method proposed in this article is not only technically feasible, but also highly accurate and reliable in practical applications, and has important practical value for accurate wheat plant morphology measurement and analysis.

## Discussion

### High-quality data collection

In this paper, we use the self-developed multi-view image acquisition system to obtain multi-view images of wheat plants and accurately reconstruct the 3D point cloud of plants by using the SFM-MVS algorithm. Compared with other 3D imaging methods such as depth camera and laser scanner, the technical solution we adopt can generate point cloud data with higher accuracy and less noise, ensure the ability to capture plant structure from all angles and reduce the problem of data loss caused by plant self-occlusion as far as possible so as to obtain the structure and morphology of wheat plants completely. The multi-view image acquisition device developed by our team has the advantages of low cost, high degree of automation and fast data acquisition efficiency. It only takes 1 min to complete the multi-view image data acquisition of a single wheat plant, and it can complete the reconstruction of a group of wheat plant point clouds in 5 min in an automated and batch manner. At the same time, the lighting system of the device also ensures the uniformity and consistency of lighting and minimizes shadows.

In addition, we quickly completed data collection within 1 h after wheat sampling and timely watered when transplanting into the flowerpot to prevent water shortage and wilting of plants, which greatly maintained the original morphology of plants. The data acquisition process is carried out in a windless indoor environment to avoid the inconsistency of plant images taken from different perspectives due to plant shaking, which will affect the quality of point cloud reconstruction. These methodological advantages lay a solid foundation for the subsequent steps of instance segmentation, 3D structure reconstruction and plant morphological parameters extraction.

### Point cloud deep learning segmentation

In this section, we mainly discuss the method of point cloud segmentation of wheat using the deep learning network. In view of the large number and overlapping characteristics of wheat leaves, we use the point cloud instance segmentation model to directly extract a single leaf instance, and its efficiency and accuracy are significantly better than the traditional process of semantic segmentation and clustering. On the test set, this model achieved a high accuracy of segmentation. For wheat plant point clouds that have not been manually edited to remove noise points, the segmentation effect is also good. These results confirmed that the model was robust with different wheat plants. Compared with the Pattern-Net wheat point cloud segmentation model (Ghahremani *et al.* 2021), our method not only achieves segmentation of wheat ear point clouds but also achieves instance segmentation of stems and leaves. In addition, during the training process, it was found that the offset loss has a large relationship with the scale of the point cloud. Reducing the scale of the point cloud will reduce the loss, but it has no significant impact on the final segmentation performance of the model. At present, our model also has some shortcomings, and the performance of the model in tiller instance segmentation needs to be improved. After analysis, the reasons are as follows: (i) The training samples are unbalanced: In the training data, the number of point clouds of tillers is far less than that of leaves, which may lead to the lack of sufficient tiller training samples in the process of tiller segmentation. (ii) Deficiency of 3D reconstruction: The occlusion of leaves may lead to missing or sparse point clouds in the process of reconstruction, thus affecting the quality of segmentation. (iii) Accuracy of data annotation: Due to the mutual adhesion of different leaf and tiller point clouds, the data annotation process is relatively difficult, and there may be a few annotation errors. (iv) Diversity of wheat growth stages: The changes in tiller structure and tiller length of wheat in different growth stages will challenge the accuracy of instance segmentation.

### 3D reconstruction and plant morphological parameters extraction

In this section, we mainly discuss the 3D reconstruction of wheat plants and the extraction of plant morphological parameters based on the segmentation results of the deep learning network, including tiller skeleton extraction, leaf skeleton extraction, leaf length, leaf width, stem leaf angle, leaf implantation site, tiller number and the tiller to which the leaf belongs. The accuracy evaluation of the extraction results shows that our method has a highly linear correlation between the predicted value and the actual value, which is acceptable for practical application.

**Figure 10.**
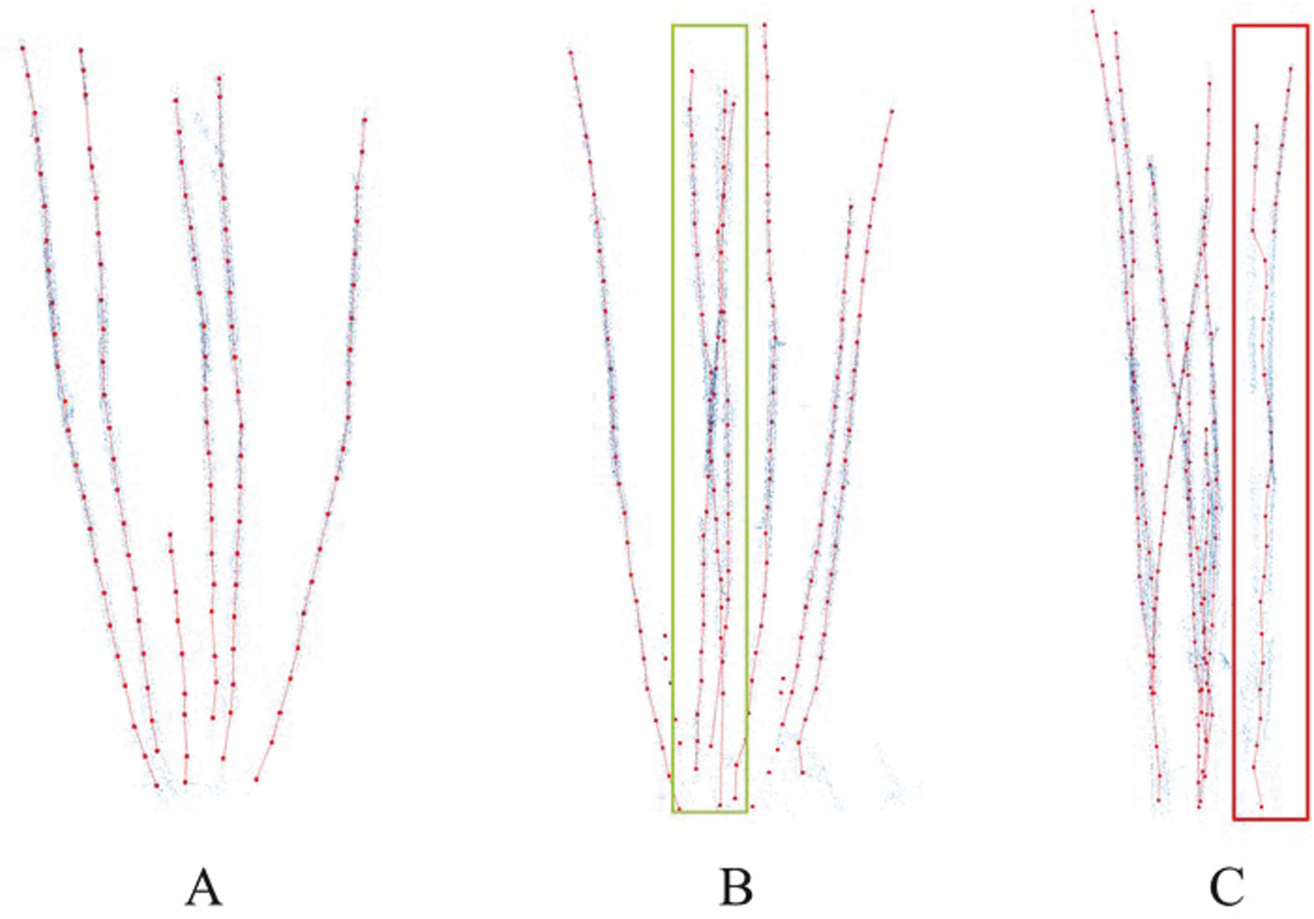
Results under different overlap degrees of tillers. (A) The Tiller skeleton is correct when there is no overlap. (B) The tiller skeleton correctly extracted when partially overlapping, the box is the overlapping part (C) The tiller skeleton cannot be correctly extracted when most of the tillers overlap, the overlapping part is in the box.

For results with different overlaps of tillers: this method works well if there is no overlap at all. If there is a small amount of cross-over overlap between different tillers, this method can reduce the impact of overlap on the results. The results will not be good if there is a lot of overlap between two tillers, causing two tillers to be recognized as one. The results are shown in Fig. 10.

**Figure 11.**
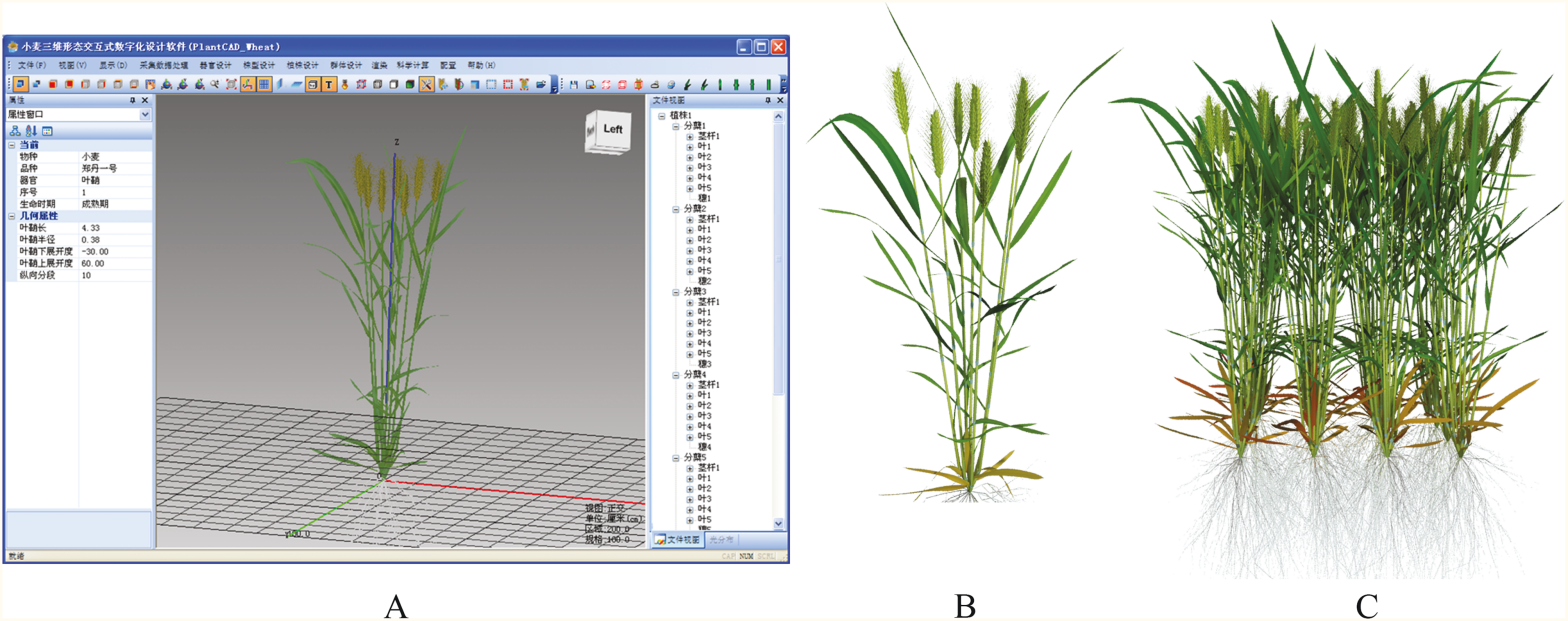
3D parametric modelling of wheat plants based on PlantCAD software series. (a) PlantCAD software main interface; (b) reconstructed individual plant of wheat; and (c) reconstructed population of wheat.

In addition, the work of this article is integrated into the PlantCAD series software (Lu S *et al.* 2014). As a plug-in, it realizes 3D parametric modelling of wheat plants driven by real-time data, replacing manual measurement and interactive design, as shown in Fig. 11.

However, we also note that there is room for improvement in the accuracy of leaf width measurement. Specific challenges include: (i) Impact of noise points: The leaf’s width is small, and it is easily affected by noise points generated during the 3D reconstruction process of the point cloud during measurement. Such outliers may make the measurement results of some leaf widths larger. (ii) Changes in leaf morphology: The leaf may curl rather than remain flat. Since the measurement of leaf width depends on the shortest straight-line distance between the two edges of the leaf, curl may cause the measured value to be smaller. In the subsequent leaf width measurement, more consideration can be given to the natural shape of the leaf to more accurately reflect the actual width of the leaf so as to improve the accuracy.

It should be pointed out that this study mainly focused on the morphological structure of leaves and tillers, only extracted some basic geometric parameters of wheat ears, and did not conduct further research on their morphological structure. In the next work, we will pay more attention to the morphological structure of wheat ears, such as ear type and ear density.

### Operation efficiency

In this paper, we use the deep learning model to optimize the instance segmentation process of wheat plant point clouds, which greatly improves the processing speed. For a single plant, the average segmentation time is only 1.7 s. In addition, the whole process, from segmentation to plant morphological parameters extraction, takes only 5.1 s. In addition, our algorithm processing flow is fully automated without any manual intervention, further reducing the operation complexity and time consumption. This fast and automatic processing capability makes the algorithm an ideal choice for processing large quantities of point cloud data. It can not only accurately reconstruct the 3D skeleton of wheat in practical application but also has high efficiency in extracting related plant morphological parameters. Compared with the 3D digitizer method (Chenxi Z *et al.* 2022), our method has greatly improved the labour cost and time efficiency of data acquisition. In the scenario of large-scale data set processing and high-throughput analysis, our algorithm shows strong competitiveness and application potential and provides an efficient solution for accurate extraction and rapid analysis of wheat plant structure information.

## Summary

In this paper, we propose a method for wheat 3D reconstruction and plant morphological parameter extraction driven by point cloud data. We achieve segmentation of wheat organs through deep learning methods and realize wheat 3D reconstruction and plant morphological parameters extraction based on segmentation results. Through the validation of wheat datasets of multiple varieties and different growth stages, our method shows excellent robustness. In addition, the wheat model reconstructed by our method and the comparative analysis of the extracted plant morphological parameters and the measured values confirmed that there was a significant correlation between them, which showed the accuracy of this method in the 3D data processing and plant morphological parameters extraction of wheat plants. We provide an effective method for 3D morphological structure reconstruction and phenotypic analysis of wheat plants, as well as an effective method for wheat agricultural production and breeding research. Future work will explore the application potential of the framework in a wider range of crop species and continue to optimize its accuracy so as to better serve projects such as precision agriculture and crop breeding.

## Data Availability

The data and code used in this article are available on GitHub, at https://github.com/lwlwr99/reconstruct-the-3D-morphological-structure-of-wheat
